# Globale Mundgesundheit im internationalen gesundheitspolitischen Rampenlicht – Herausforderungen und neue Chancen für nachhaltige Verbesserungen

**DOI:** 10.1007/s00103-021-03353-6

**Published:** 2021-06-08

**Authors:** Habib Benzian, Stefan Listl

**Affiliations:** 1 Department of Epidemiology & Health Promotion, WHO Collaborating Center for Quality Improvement & Evidence-based Dentistry, College of Dentistry, New York University, New York, USA; 2 Lehrstuhl für Quality and Safety of Oral Health Care, Department of Dentistry, Radboud University Medical Center, Radboud Institute for Health Sciences, Nijmegen, Niederlande; 3 Sektion Translationale Gesundheitsökonomie, Universitätsklinikum Heidelberg, Poliklinik für Zahnerhaltungskunde, Im Neuenheimer Feld 400, 69120 Heidelberg, Deutschland

**Keywords:** Zahnmedizinische Versorgung, Globale Krankheitslast, Gesundheitspolitik, Karies, Politische Priorisierung, Oral health care, Global burden of disease, Health policy, Dental caries, Political priority

## Abstract

Orale Erkrankungen sind ein signifikantes globales Gesundheitsproblem über alle Länder und Bevölkerungsgruppen hinweg. Mit fast 3,5 Mrd. Erkrankungsfällen (2017) sind so viele Menschen betroffen wie von keiner anderen Krankheitsgruppe. Die Haupterkrankungen sind unbehandelte Karies der bleibenden oder der Milchzähne, fortgeschrittene Parodontopathien, Zahnlosigkeit sowie Karzinome der Mundhöhle und Lippen. Bei weitgehend unverändert hoher globaler Prävalenz erhöhen durch Bevölkerungswachstum bedingte stark steigende Fallzahlen den Druck auf schwache oder überlastete Gesundheitssysteme, insbesondere in Ländern der unteren und mittleren Einkommensgruppen.

Dennoch wird der Mundgesundheit in vielen Ländern nur unzureichende Priorität eingeräumt und sie erhält als wichtiges Thema im gesundheitspolitischen Diskurs der deutschen und globalen Akteure nach wie vor wenig Aufmerksamkeit. Eine der fundamentalen Herausforderungen ist dabei die Gewährleistung eines allgemeinen und fairen Zugangs zu adäquater universeller Basisgesundheitsversorgung für alle Menschen ohne Verursachung von finanziellen Härten (Universal Health Coverage).

Dieser Beitrag gibt einen einführenden Überblick über die globalen Trends der weltweiten Krankheitslast der oralen Haupterkrankungen, die von starken Ungleichheiten geprägt sind. Verbesserungsansätze aus der bevölkerungsweiten Risikoreduktion und Prävention, der Versorgungsplanung sowie gesundheitspolitische Lösungen werden kurz vorgestellt. Dabei werden die im internationalen Diskurs wichtigen Themen angesprochen und die im Rahmen einer *Lancet*-Artikelserie zur globalen Mundgesundheit aus dem Jahr 2019 entwickelten Reformbereiche besprochen. Schließlich werden neue Initiativen diskutiert sowie Empfehlungen für die deutsche und internationale gesundheitliche Entwicklungspolitik gegeben, die in den kommenden Jahren die Situation der globalen Mundgesundheit entscheidend verbessern könnten.

## Abstract (en)

Oral diseases are a significant global health problem across all countries and populations. With about 3.5 billion cases (2017), more people are affected than by any other disease group. The main oral diseases comprise tooth decay of permanent and deciduous teeth, severe periodontal disease, and oral and lip cancer. With a largely unchanged high global prevalence, but significantly growing population sizes, the pressure on health systems is increasing, particularly in low- and middle-income countries.

Nonetheless, in many countries oral health has insufficient priority as a key health topic, including the global health policy discourse of German and international stakeholders. One of the fundamental challenges is ensuring universal and equitable access to basic oral healthcare services for all and without financial hardship (Universal Health Coverage).

This paper provides an introductory overview of the global trends for the main oral diseases, which are generally characterized by stark inequalities. Opportunities for improving the situation through population-wide risk reduction and preventive approaches, access to oral healthcare, and policy options are highlighted. In addition, a range of relevant global (oral) health topics with potential for tangible change are discussed. Lastly, the reform areas of the Lancet Series on Oral Health from 2019 are presented and recommendations for the German and international global health policy discourse are provided.

## Fortschritte in der Mundgesundheit im globalen Kontext?

Die Situation der Mundgesundheit ist ein globales Gesundheitsproblem über alle Länder und Bevölkerungsgruppen hinweg. Mit geschätzten fast 3,5 Mrd. Erkrankungsfällen weltweit 2017 sind so viele Menschen betroffen wie von keiner anderen Krankheitsgruppe. Auch wenn sich die Herausforderungen in Ländern der hohen Einkommensgruppe (Weltbankklassifikation) deutlich von den Krankheits- und Versorgungsproblemen in Ländern der niedrigen oder mittleren Einkommensgruppen unterscheiden, so gibt es doch viele Parallelen, grundlegende globale Probleme und auch globale Lösungsansätze, die lokal umgesetzt werden können. Eine der fundamentalen Herausforderungen ist dabei die Gewährleistung eines allgemeinen und fairen Zugangs zu adäquater universeller Basisgesundheitsversorgung für alle Menschen und ohne finanzielle Härten. Dieses im Englischen auch als Universal Health Coverage (UHC) bezeichnete Konzept ist Teil der von der Weltgesundheitsorganisation (WHO) in den Nachhaltigkeitszielen der Vereinten Nationen verankerten Entwicklungsziele bis zum Jahr 2030, auch „Agenda 2030“ genannt [1].

Leider wird der Zahn- und Mundgesundheit in vielen Ländern nur unzureichende gesundheitspolitische Priorität eingeräumt. Dies trifft insbesondere in den Ländern zu, deren öffentliche Gesundheitsausgaben eher gering sind und die dadurch kaum eine essenzielle Gesundheitsversorgung der Bevölkerung gewährleisten können. Obwohl die enge Verknüpfung zwischen oraler und allgemeiner Gesundheit sowie die gesellschaftliche Relevanz der Mundgesundheit hinreichend belegt sind, erhält die Mundgesundheit als wichtiges Gesundheitsthema und Interventionsbereich im allgemeinen politischen Diskurs der deutschen und globalen Akteure nach wie vor wenig Aufmerksamkeit [2].

Dieser Beitrag stützt sich in weiten Teilen auf die Analysen und Empfehlungen der Artikelserie zur Mundgesundheit, die 2019 erstmals in der renommierten medizinischen Fachzeitschrift *The Lancet* erschienen ist und an der beide Co-Autoren aktiv beteiligt waren [3]. Zunächst werden die globalen Trends der seit 30 Jahren fast stetig ansteigenden Krankheitslast für die oralen Haupterkrankungen dargestellt und einige der vielschichtigen und komplexen Ursachen für diese Situation beleuchtet. Schließlich werden Ansätze aus der bevölkerungsweiten Risikoreduktion und Prävention, der Versorgungsplanung sowie gesundheitspolitische Lösungen vorgestellt. Empfehlungen für die deutsche und internationale gesundheitliche Entwicklungspolitik, die in den kommenden Jahren die Situation der globalen Mundgesundheit entscheidend verbessern könnten, schließen den Beitrag ab.

## Globale Krankheitslast und Ungleichheiten der oralen Haupterkrankungen

**Figure 1:**
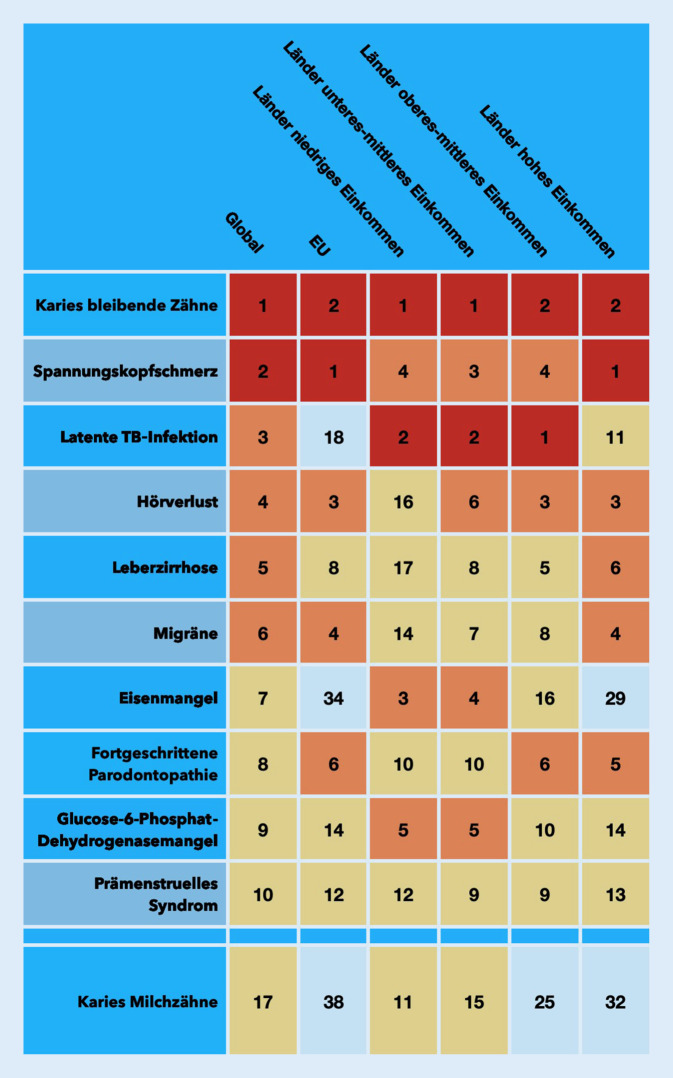

Unbehandelte Karies der bleibenden oder der Milchzähne, fortgeschrittene Parodontopathien, Zahnlosigkeit sowie Karzinome der Mundhöhle und Lippen machen den Großteil der weltweit ungefähr 3,5 Mrd. Fälle von oralen Erkrankungen aus (2017; [4]). Die globale epidemiologische Einschätzung der Mundgesundheit hat sich in den letzten Jahren im Zuge verbesserter Modellrechnungen erheblich gewandelt. Maßgeblich dazu beigetragen haben die regelmäßig erscheinenden *Global Burden of Disease Studies *des Institute of Health Metrics and Evaluation in Seattle (USA). Dabei wird zunehmend deutlich, dass sich die Prävalenzen der oralen Haupterkrankungen seit 1990 kaum verändert haben. Die Fallzahlen betroffener Menschen hingegen haben durch das demografische Wachstum der Weltbevölkerung erheblich zugenommen, insbesondere in Ländern der niedrigen und mittleren Einkommensgruppen [4–6]. Die stark gestiegene Anzahl von Menschen mit unbehandelten oralen Erkrankungen stellt so eine erhebliche Belastung für schwache oder ohnehin schon überforderte Systeme der zahnmedizinischen Versorgung dar. Abb. 1 gibt eine Übersicht zu den 10 häufigsten Erkrankungen (plus Milchzahnkaries) weltweit, sortiert nach ihrer Prävalenz und für verschiedene Ländergruppen (beide Geschlechter, alle Altersgruppen, 2017; [7]).

Unbehandelte Karies der bleibenden Zähne ist die häufigste Krankheit der Menschheit mit geschätzten 2,3 Mrd. Fällen. Unbehandelte Milchzahnkaries steht an 17. Stelle in der Häufigkeit aller Erkrankungen, mit geschätzten 530 Mio. Fällen (2017; [4]). Lediglich in Ländern der hohen Einkommensgruppe haben die Prävalenz und der Schweregrad von Karies beider Dentitionen im Zeitraum 1990–2017 abgenommen. In allen anderen Ländern hingegen ist die Prävalenz jedoch leicht gestiegen, sodass die globale Prävalenz in diesem Zeitraum so gut wie unverändert geblieben ist. Durch den allgemeinen Bevölkerungszuwachs ist allerdings die Zahl der Fälle (und damit die Zahl der versorgungsbedürftigen Menschen) erheblich gestiegen, am stärksten in Ländern der mittleren Einkommensgruppe [2].

Fortgeschrittene Parodontopathien stehen in der weltweiten Häufigkeit aller Erkrankungen an 8. Stelle mit fast 800 Mio. geschätzten Fällen, in Ländern der hohen Einkommensgruppe sogar an 5. Stelle (2017). Dabei zeigen sich die größten Zuwächse in Ländern der niedrigen Einkommensgruppe und die höchsten Fallzahlen in Ländern der mittleren Einkommensgruppe [4].

Als Endpunkt von lebenslangen Erkrankungen wie Karies oder Parodontopathien betrifft komplette Zahnlosigkeit weltweit fast 270 Mio. Menschen, vorwiegend im höheren Alter. Die Prävalenz ist in Ländern der niedrigen Einkommensgruppe am geringsten, dort und in Ländern der unteren bis mittleren Einkommensgruppe ist sie jedoch im Zeitraum 1990–2017 am stärksten angestiegen [4].

Zuverlässige epidemiologische Daten zu Karzinomen der Mundhöhle und Lippen (ICD-Codes C00-06) wurden kürzlich von der International Agency for Research on Cancer (IARC), einer Unterorganisation der WHO, vorgestellt [8]. Die Gruppe dieser Krebserkrankungen steht an 16. Stelle aller Krebserkrankungen mit geschätzten 378.000 neuen Fällen, fast 180.000 Todesfällen pro Jahr und einer 5‑Jahres-Prävalenz von fast 960.000 Fällen. Die altersstandardisierte Inzidenz ist am höchsten in Melanesien (16,7/100.000), Süd‑/Zentralasien (9/100.000), gefolgt von Australien und Neuseeland (6/100.000) und Zentral‑/Osteuropa (5,1/100.000) mit jeweils erheblich höheren Raten für Männer. In Ländern wie Indien oder Pakistan ist Mundhöhlenkrebs die häufigste Todesursache [9]. Risikofaktoren wie Tabak- und Alkoholkonsum spielen die größte Rolle in der Krebsentstehung sowie kulturspezifische Praktiken wie Betelnusskonsum. Verstärkte Evidenz für humane-papillomviren-(HPV-)assoziierte orale Karzinome hat in vielen Ländern eine Anpassung von Impfempfehlungen beschleunigt [10, 11].

Epidemiologische Daten für andere orale Erkrankungen, wie orofaziales und dentales Trauma, orofaziale Spalten, Mundschleimhauterkrankungen oder Noma (Wangenbrand) sind unvollständig oder werden gar nicht koordiniert erhoben. Als Folge lässt sich deren Relevanz für die Bevölkerungsgesundheit nur schwer abschätzen (mit Ausnahme von Spalterkrankungen; [12–14]).

Alle oralen Erkrankungen sind von deutlichen Ungleichheiten geprägt. Benachteiligte Bevölkerungsgruppen, Menschen mit niedrigem sozioökonomischem Status, niedrigerer Bildung, Migranten, Wohnungslose, ethnische Minderheiten oder Populationen, die aus anderen Gründen ausgegrenzt sind, leiden generell verstärkt unter oralen Erkrankungen und haben oftmals gleichzeitig schlechteren Zugang zu adäquater zahnmedizinischer Versorgung und Präventionsmaßnahmen [2]. Dabei zeigt sich deutlich der Einfluss von sozialen Gesundheitsdeterminanten. Auch die negative Rolle von allgemeinen gesundheitlichen Risikofaktoren wird sichtbar. Dazu zählen Tabakkonsum, übermäßiger Alkoholgebrauch, ungesunde und stark zuckerhaltige Ernährung, mangelnde Hygiene, um nur die wichtigsten Elemente der sogenannten „common risk factors“ zu nennen [2]. Die Kontrolle dieser Risikofaktoren, die für die oralen Haupterkrankungen und alle anderen chronischen Erkrankungen gleichermaßen verantwortlich sind, ist einer der vielversprechendsten Ansätze in der Prävention und bevölkerungsweiten Gesundheitsförderung [15].

## Weltweite Kosten und wirtschaftliche Auswirkungen oraler Erkrankungen

Um die wirtschaftlichen Auswirkungen oraler Erkrankungen und der zahnmedizinischen Versorgung auf Einzelpersonen und die gesamte Gesellschaft zu verstehen, ist es hilfreich, verschiedene Kostenkategorien zu betrachten. Dazu zählen die direkten Kosten (Versorgungskosten), indirekten Kosten (Produktivitätsverluste) und immateriellen Kosten im Sinne eingeschränkter Lebensqualität. Zahnerkrankungen haben erhebliche wirtschaftliche Auswirkungen auf Einzelpersonen und Gesellschaften [16, 17]. Weltweit entstanden im Jahr 2015 direkte Kosten in Höhe von 356,80 Mrd. USD und indirekte Kosten in Höhe von 187,61 Mrd. USD in Verbindung mit Zahnerkrankungen. Dabei entfallen ungefähr 80 % der globalen Ausgaben auf lediglich 20 % der Weltbevölkerung, vornehmlich in Ländern der hohen Einkommensgruppe. In den Ländern der Europäischen Union (EU) führten Zahnerkrankungen zu Behandlungsausgaben von ungefähr 90 Mrd. Euro und zu Produktivitätsverlusten von rund 52 Mrd. Euro im Jahr 2015 [17, 18].

**Figure 2:**
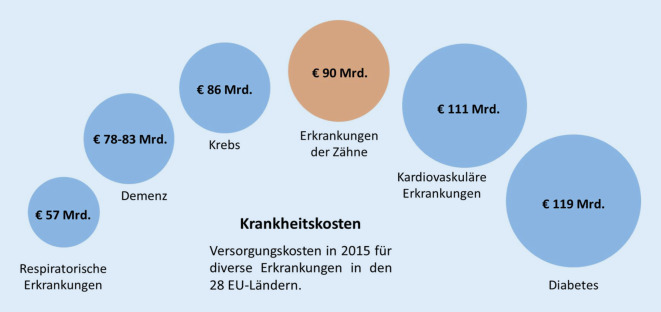

Die zahnärztlichen Ausgaben sind sowohl in absoluten Zahlen als auch im Verhältnis zu den Kosten für die Behandlung anderer Krankheiten erheblich [2]. Die direkten Kosten für Zahnerkrankungen in EU-Ländern stehen an 3. Stelle hinter den direkten Kosten für Diabetes und Herz-Kreislauf-Erkrankungen, jedoch vor Demenz, Atemwegserkrankungen und Krebs (Abb. 2; [17]). Für eine Reihe von Ländern, insbesondere Länder der niedrigen und mittleren Einkommensgruppe, wurde nachgewiesen, dass Selbstbehalte für zahnmedizinische Versorgung zu katastrophalen Gesundheitsausgaben führen und Haushalte in die Armut treiben können [19, 20].

Orale Erkrankungen können die Aktivitäten des täglichen Lebens deutlich einschränken. Erhebliche Fehlzeiten und reduzierte Leistungsfähigkeit am Arbeitsplatz und in der Schule sowie schlechtere Chancen auf dem Arbeitsmarkt und geringeres Einkommen sind nur einige der möglichen Folgen [21, 22]. Für die erwachsene US-Bevölkerung wurde geschätzt, dass Zahnerkrankungen einen Verlust an qualitätsadjustierter Lebenserwartung (sog. Quality Adjusted Life Expectancy = QALE) in Höhe von 5,3 % des QALE-Verlusts durch die Gesamtmorbidität ausmacht [23]. QALE ist ein aggregiertes Maß für die Lebenserwartung unter Berücksichtigung der Lebensqualität. Weiterhin können orale Erkrankungen die Morbidität und Versorgungskosten anderer systemischer Erkrankungen erhöhen. So wird Parodontitis mit einer schlechteren Blutzuckerkontrolle bei Diabetikern in Verbindung gebracht. Untersuchungen auf Grundlage von administrativen Routinedaten zeigen, dass die Versorgung von Parodontitis zu einer Senkung der diabetesbedingten Versorgungskosten führen kann [24, 25].

## Aktueller internationaler Diskurs zur Mundgesundheit

Im internationalen Kontext ist derzeit eine Vielzahl von Themen in der Diskussion, die im Hinblick auf eine bessere Priorisierung der zahnmedizinischen Grundversorgung für alle von Bedeutung sind oder die als Anknüpfungspunkte dienen könnten, um die Mundgesundheit in neue gesundheitspolitische Kreise einzubringen:

- Definition von essenzieller zahnmedizinischer Grundversorgung, insbesondere vor dem Hintergrund der COVID-19-Pandemie, aber vor allem um entsprechende Leistungspakete in eine universelle Basisgesundheitsversorgung zu integrieren [26, 27]
- Angesichts von Milliarden von Menschen ohne nachhaltigen Zugang zu bezahlbarer zahnmedizinischer Versorgung ist die Entwicklung und Priorisierung kosteneffizienter Interventionen zur zahnmedizinischen Grundversorgung eines der wichtigsten Themen (z. B. nach dem WHO-Konzept der *Best Buys *oder der *NCD Investment Cases*; [28])
- Verbesserte Integration von medizinischer und zahnmedizinischer Basisversorgung, insbesondere in Ländern der niedrigen und mittleren Einkommensgruppe [29]
- Ungleiche Verteilung von zahnmedizinischem Fachpersonal: Zahnmedizinische Über- und Unterversorgung existieren parallel; 69 % der ZahnärztInnen weltweit kümmern sich um die zahnmedizinische Versorgung von 27 % der Weltbevölkerung [30]. Modelle für nationale Bedarfs- und Ressourcenplanung halten mit den Veränderungen in Morbidität und medizinisch-technologischem Fortschritt nicht mit. Innovative Ansätze sollten die optimale Versorgung der Bevölkerung auf der Basis der Bedarfe, Ressourcen und möglichen Gesundheitsresultate mit besserer Präzision ermöglichen [31]
- Entwicklung neuer, kosteneffektiver Füllungswerkstoffe. Dies ist insbesondere im Hinblick auf das mittelfristige Ende der Nutzung von Amalgam relevant, das im Rahmen der Minamata-Konvention der Vereinten Nationen zur Eliminierung von Quecksilber aus der Umwelt beschlossen wurde [32]
- Verbesserung der zahnmedizinischen Verschreibungspraxis von Antibiotika und Integration in globale Strategien gegen antimikrobielle Resistenzen [33]
- Verstärkter Fokus auf Klima- und Umweltschutz zur Verbesserung der Nachhaltigkeit in der Zahnmedizin [34, 35]
- Kritische Diskussion um Chancen und Herausforderungen des zunehmenden Einsatzes von Digitalisierung, Telezahnmedizin, Big Data und künstlicher Intelligenz [36]

Diese kurze und unvollständige Auflistung relevanter Themenbereiche zeigt einerseits die Breite der inhaltlichen Diskussionen. Sie ist aber auch charakteristisch für einen erheblichen Nachholbedarf in der zahnmedizinischen und Dental-Public-Health-Debatte, Forschung, Programmplanung/-implementierung und Evaluierung. Die jahrzehntelange separate Betrachtung der Mundgesundheit und allgemeiner Gesundheit auf wissenschaftlicher, klinischer und politischer Ebene hat in vielen Bereichen zu einer Abkopplung vom internationalen gesundheitlichen und gesundheitspolitischen Diskurs geführt. Die geringe Aufmerksamkeit und mangelhafte Priorisierung der globalen Mundgesundheit sind die offensichtlichen Resultate dieser Fehlentwicklung.

## Eine neue Vision für die globale Mundgesundheit

Die *Lancet*-Serie zur Mundgesundheit greift viele der dargestellten Herausforderungen und Chancen auf und bündelt sie zu 8 Reformempfehlungen, die gemeinsam das Potenzial für nachhaltige Verbesserung der globalen Mundgesundheit haben [15].

1. Bessere internationale Daten
  Die Verfügbarkeit von standardisierten epidemiologischen Daten zur Mundgesundheit als Teil der Routinegesundheitsüberwachung muss gestärkt werden, idealerweise integriert in die Erhebung von Daten zu chronischen Erkrankungen. Ebenso wichtig sind Indikatoren und Daten zu Gesundheitssystemen, um eine Integration in Konzepte der universellen Basisgesundheitsversorgung zu unterstützen, aber auch patientenzentrierte Informationen zur Versorgungsqualität [37]
2. Reform von zahnmedizinischen Versorgungsmodellen
  Das Ziel der universellen Gesundheitsversorgung erfordert die Entwicklung von Versorgungspaketen mit guter Kosteneffizienz für die häufigsten Mundkrankheiten, um Regierungen im Rahmen einer Basisgesundheitsversorgung versorgungspolitische Optionen zu eröffnen und die Integration in den größeren Kontext der Bekämpfung von chronischen nichtübertragbaren Erkrankungen möglich zu machen. Dazu hat die WHO eine Rahmenmethodik entwickelt, mit der die Kosteneffizienz von Interventionen vergleichbar evaluiert werden kann (WHO „Best buys for prevention and control of noncommunicable diseases“), die noch auf die prioritären zahnmedizinischen Interventionen angewendet werden muss. Bezahlungs- und Kostenerstattungssysteme sollten Anreize für ganzheitliche Behandlung, Prävention und unterstützende Begleitung setzen und die vorherrschenden Einzelleistungsprinzipien ablösen [27]
3. Anpassungen in der Ausbildung des Gesundheitspersonals
  Die Reform von Versorgungsmodellen sollte durch eine Reform der Ausbildung des beteiligten Gesundheitspersonals ergänzt und unterstützt werden. Das bisherige, auf die zentrale Rolle des Zahnarztes/der Zahnärztin ausgerichtete Personal- und Führungskonzept muss dabei von einem Teammodell abgelöst werden, das flexibel auf verfügbare Ressourcen und Bevölkerungsbedarfe eingestellt werden kann, um angepasste, evidenzbasierte und hochqualitative zahnmedizinische Versorgung zu gewährleisten
4. Adressierung von Ungleichheiten in der Mundgesundheit
  Reduzierung der erheblichen Ungleichheiten in der Krankheitslast und im Zugang zu zahnmedizinischer Versorgung sollte Priorität haben und durch inklusive, niederschwellige, erschwingliche und leicht zugängliche Angebote gefördert werden. Alle Aspekte der Personalausbildung und Versorgung sollten an diesen Prinzipien ausgerichtet werden und dabei die außerhalb des Gesundheitssystems angesiedelten Determinanten mit einbeziehen
5. Schwerpunktsetzung auf systemische bevölkerungsweite Gesundheitsförderung
  Der momentane Schwerpunkt von Prävention liegt vornehmlich auf individuellen, klinischen oder erzieherischen Maßnahmen, die lediglich kurzfristige Erfolge zeigen. Ein systemischer Ansatz der bevölkerungsweiten Gesundheitsförderung, der die zugrunde liegenden Ursachen und Risiken von oralen Erkrankungen ins Visier nimmt, wäre erheblich wirkungsvoller und kosteneffektiver. Zusätzlich kann von einer Hebelwirkung für andere chronische Erkrankungen, die durch die gleichen Risikofaktoren verursacht werden, ausgegangen werden
6. Eindämmung von kommerziellen Gesundheitsdeterminanten
  Gesundheitsschädliche kommerzielle Interessen bestimmter Industriezweige (z. B. Tabak‑, Zucker- und Lebensmittelindustrie) müssen durch wirksame politische Steuerungsmaßnahmen eingedämmt werden, um insbesondere vulnerable Bevölkerungsgruppen zu schützen. Dazu zählen Werbeverbote, selektive Steuererhöhungen und andere fiskale Maßnahmen oder Regelungen für gesunde Schulernährung, um nur einige Beispiele zu nennen [38]. Wichtig sind ebenfalls Transparenz und Offenlegung von Interessenskonflikten, um unangemessene Einflussnahme der Industrie auf Politikgestaltung, Forschung und den zahnärztlichen Berufsstand zu verhindern [39, 40]
7. Evolution der zahnmedizinischen Forschungsansätze
  Eine koordinierte internationale Forschungsagenda mit klarer Priorität für Implementierungs- und Gesundheitssystemforschung, insbesondere in Entwicklungsländern ist vonnöten, um den Anschluss an andere medizinische Themen nicht zu verlieren. Dabei sollten verstärkt interdisziplinäre und auch qualitative Methoden zum Einsatz kommen, um den bisherigen Schwerpunkt auf klinischer Forschung in Richtung auf die Schließung wichtiger Evidenzlücken in der populationsbasierten Versorgungsforschung zu verschieben [41, 42]
8. Verstärkte Lobbyarbeit für bessere gesundheitspolitische Priorität
  Die Vernachlässigung der Mundgesundheit im Kontext der internationalen Gesundheitspolitik sollte durch verstärktes Lobbying und politische Überzeugungsarbeit angegangen werden [43]. Die Rolle der Mundgesundheit im Hinblick auf die Erreichung der nachhaltigen Entwicklungsziele der Vereinten Nationen oder im Zusammenhang mit dem globalen Aktionsplan der WHO zur Eindämmung der chronischen Krankheiten sind dabei wichtige argumentative Anknüpfungspunkte, um die gesundheitspolitische Priorität der Mundgesundheit zu stärken

Auf dem Weg zur Realisierung dieser Empfehlungen wurden bereits einige wichtige erste Schritte gemacht und weitere werden folgen. Die Mundgesundheit konnte als gesundheitlicher Aktionsbereich für Regierungen in 2 wichtigen politischen Deklarationen der Vereinten Nationen verankert werden, die so eine wichtige argumentative Basis zur Politikgestaltung und Versorgungsplanung im Kontext von chronischen Erkrankungen und allgemeiner Gesundheitsversorgung bieten [27, 44, 45]. Im Januar 2021 wurde vom Exekutivrat der WHO eine Resolution verabschiedet, mit der die WHO aufgefordert wird, eine globale Strategie und einen Aktionsplan für Mundgesundheit 2022–2031 zu erarbeiten, einschließlich eines globalen Monitoringsystems [46, 47]. Zusammen mit dem Bericht des US-amerikanischen Surgeon General (Direktor des öffentlichen Gesundheitsdienstes der USA) zum Thema Mundgesundheit und dem Report der WHO zur Weltmundgesundheit, deren Veröffentlichung in diesem Jahr vorgesehen ist, wird das Thema Mundgesundheit somit erstmals seit langer Zeit wieder im internationalen gesundheitspolitischen Rampenlicht stehen.

## Entwicklungspolitische Empfehlungen für globale Mundgesundheit

Seit Jahren beklagen die WHO und andere Organisationen die massive Unterfinanzierung der Prävention und Kontrolle chronischer, nichtübertragbarer Erkrankungen, insbesondere im Kontext von Entwicklungszusammenarbeit mit Ländern der unteren und mittleren Einkommensgruppe [48]. Lediglich weniger als 2 % der Unterstützungsausgaben für Gesundheit sind dieser Krankheitsgruppe zugeordnet, obwohl 4 von 5 Menschen mit chronischen Krankheiten in diesen Ländern leben. Auch als Teil der chronischen Krankheiten ist die Mundgesundheit bisher im Bereich der Entwicklungszusammenarbeit überhaupt nicht sichtbar, was auch daran liegen mag, dass große Organisationen (z. B. UNICEF) das Thema noch nicht als Problem für die Gesundheit von Kindern erkannt haben. Es ist zu hoffen, dass die verstärkten Anstrengungen der WHO, das Thema auf die globale gesundheits- und entwicklungspolitische Agenda zu heben, zu einer Umsteuerung führen werden. Der dringend notwendige massive Investitionsanstieg für chronische Erkrankungen muss dabei die Stärkung der Mundgesundheit als Teil der Gesundheitssystemstärkung miteinschließen und so die Synergien einer guten Regierungsführung (Good Governance) und nachhaltigen Gesundheitsförderung für alle chronischen Erkrankungen nutzen (z. B. durch Besteuerung von gesundheitsschädlichen Lebensmitteln und Produkten, Tabakkontrolle). Die deutsche Entwicklungszusammenarbeit hat mit dem von der Gesellschaft für Internationale Zusammenarbeit (GIZ) GmbH im Auftrag des Bundesministeriums für wirtschaftliche Zusammenarbeit und Entwicklung implementierten Fit-for-School-Ansatz ein international anerkanntes Programmkonzept zur Verfügung gestellt. Der Ansatz zielt darauf ab, durch intersektorale Zusammenarbeit die Gesundheit und das Wohlbefinden von SchülerInnen zu verbessern. Dabei geht es vor allem um die Verbesserung des Zugangs zu sauberem Wasser sowie die Sanitär- und Hygieneversorgung in Schulen als Voraussetzung für die Schaffung eines gesundheitsförderlichen Umfeldes. Dadurch wird die Etablierung von täglichen Hygienemaßnahmen u. a. zur Verbesserung von Mundgesundheit durch tägliches Zähneputzen mit fluoridhaltiger Zahnpasta erst möglich [49, 50]. Die Bundesregierung ist aufgefordert, ihr Engagement für multilaterale Organisationen wie die WHO zu verstärken und dabei ihre führende internationale Rolle auch im Kontext der globalen Mundgesundheit einzubringen.

## Schlussbetrachtungen

Gesundheit ist ein fundamentales Menschenrecht und gute Mundgesundheit ist als integraler Teil dieses Grundrechts anerkannt. Die universale Realisierung dieses Menschenrechts ist jedoch noch in weiter Ferne. Milliarden Menschen haben weiterhin keinen nachhaltigen und bezahlbaren Zugang zu Basisgesundheitsdiensten, einschließlich Grundleistungen einer zahnärztlichen Primärversorgung oder Prävention. Den Ländern der hohen Einkommensgruppe kommt der Verbesserung dieser Situation eine besondere Verantwortung zu, die auch den zahnärztlichen Berufsstand als Ganzes miteinschließt. Die simple Forderung nach mehr zahnärztlicher Ausbildung in Entwicklungsländern greift dabei zu kurz und verkennt die Komplexität der notwendigen Maßnahmen zur Stärkung lokaler Gesundheitssysteme. Der Transfer von technologiefokussierten und kurativen zahnärztlichen Behandlungsansätzen sollte daher durch eine bedarfsgerechte, an vorhandene Ressourcen angepasste und innovative Planung ersetzt werden, bei der auch moderne Konzepte der populationsbasierten Prävention sowie die Einbindung anderer Berufsgruppen des Gesundheitssystems im Vordergrund stehen [15, 31]. Das vom Weltzahnärzteverband FDI kürzlich vorgestellte Konzept einer Vision 2030 beschreibt einige der dabei notwendigen Anpassungen und betont die Notwendigkeit von lokalen Lösungen unter Einbindung der Zivilgesellschaft [33]. Selbstverständlich sind die weiterhin bestehenden Ungleichheiten und die erheblich höheren Krankheitslasten benachteiligter Bevölkerungsgruppen in Ländern der hohen Einkommensgruppe besorgniserregend und müssen durch proaktive Politiken sowie gruppenspezifische Präventions- und Versorgungsangebote adressiert werden.

Somit bleibt die globale Mundgesundheit auf absehbare Zeit ein wichtiges Problem der internationalen Gesundheitspolitik und der öffentlichen Gesundheit in allen Ländern. Nur durch gemeinsame Anstrengungen auf allen Ebenen – Public Health, Gesundheitssysteme, Prävention, klinische Versorgung, Finanzierung, Forschung und Ausbildung – werden nachhaltige Verbesserungen möglich sein. Die Stärkung von politischer Priorität und Sichtbarkeit des vermeintlichen Nischenthemas der globalen Mundgesundheit bleibt dabei einer der wichtigsten Ansatzpunkte.
